# Supplementary material for: Novel Driver Strength Index highlights important cancer genes in TCGA PanCanAtlas patients
Source: PeerJ. 2022 Aug 11;10:e13860. doi: 10.7717/peerj.13860 (PMC9375969; doi:10.7717/peerj.13860)
Supplement: Supplemental Information 2 [file peerj-10-13860-s002.zip › COHORTS/genes plots/cohorts/2021_8_16_14_9_distribution_cohorts_males_COAD.pdf]

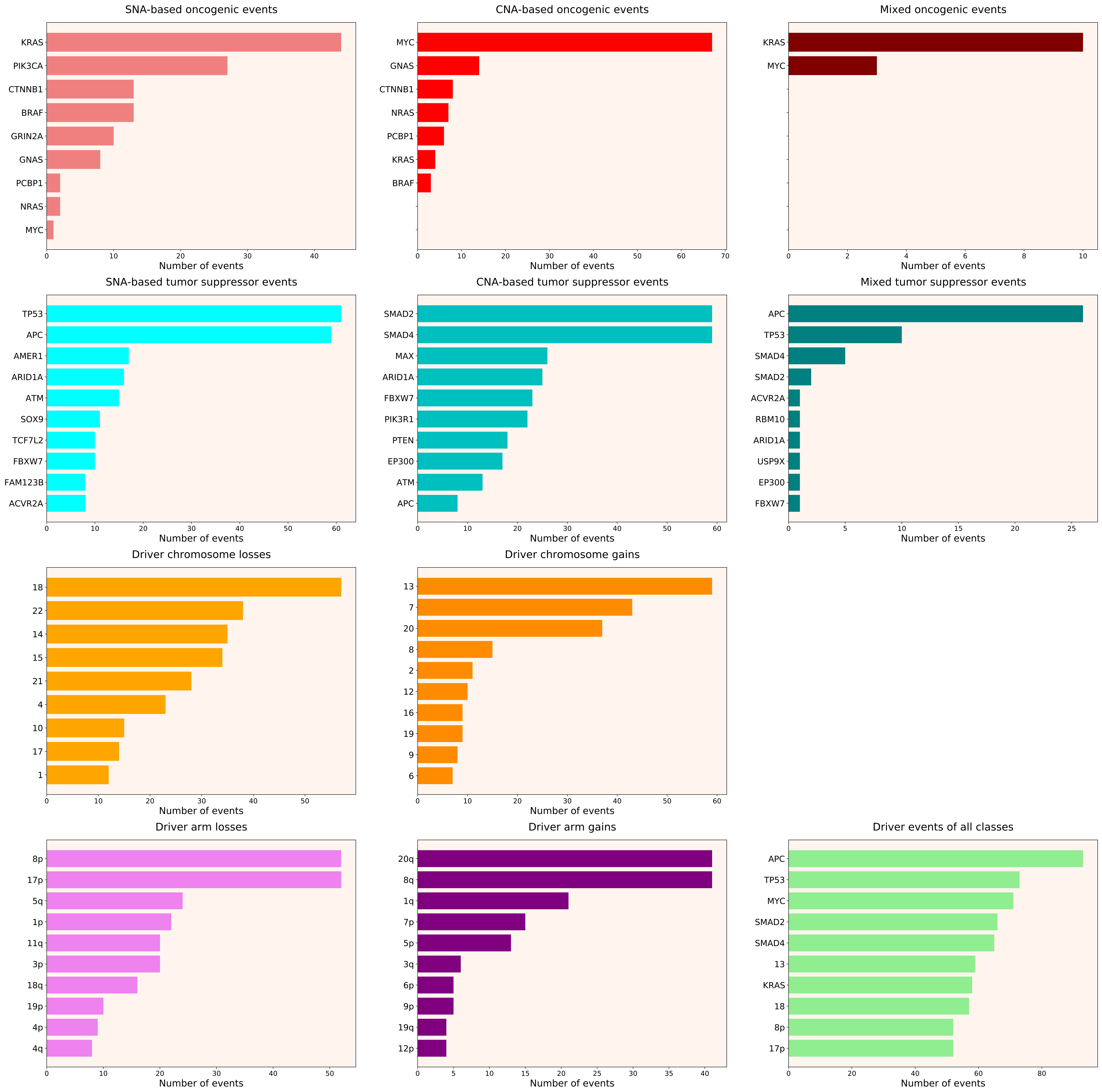
SNA-based oncogenic events
KRAS
PIK3CA
CTNNB1
BRAF
GRIN2A
GNAS
PCBP1
NRAS
MYC
Number of events
CNA-based oncogenic events
MYC
GNAS
CTNNB1
NRAS
PCBP1
KRAS
BRAF
Number of events
Mixed oncogenic events
KRAS
MYC
Number of events
SNA-based tumor suppressor events
TP53
APC
AMER1
ARID1A
ATM
SOX9
TCF7L2
FBXW7
FAM123B
ACVR2A
Number of events
CNA-based tumor suppressor events
SMAD2
SMAD4
MAX
ARID1A
FBXW7
PIK3R1
PTEN
EP300
ATM
APC
Number of events
Mixed tumor suppressor events
APC
TP53
SMAD4
SMAD2
ACVR2A
RBM10
ARID1A
USP9X
EP300
FBXW7
Number of events
Driver chromosome losses
18
22
14
15
21
4
10
17
1
Number of events
Driver chromosome gains
13
7
20
8
2
12
16
19
9
6
Number of events
Driver arm losses
8p
17p
5q
1p
11q
3p
18q
19p
4p
4q
Number of events
Driver arm gains
20q
8q
1q
7p
5p
3q
6p
9p
19q
12p
Number of events
Driver events of all classes
APC
TP53
MYC
SMAD2
SMAD4
13
KRAS
18
8p
17p
Number of events
